# Irritability, Defiant and Obsessive-Compulsive Problems Development from Childhood to Adolescence

**DOI:** 10.1007/s10964-021-01528-7

**Published:** 2021-11-02

**Authors:** Lourdes Ezpeleta, Eva Penelo, J. Blas Navarro, Núria de la Osa, Esther Trepat

**Affiliations:** 1 Unitat d’Epidemiologia i de Diagnòstic en Psicopatologia del Desenvolupament, Universitat Autònoma de Barcelona, Barcelona, Spain; 2 Departament de Psicologia Clínica i de la Salut, Universitat Autònoma de Barcelona, Barcelona, Spain; 3 Departament de Psicobiologia i de Metodologia de les Ciències de la Salut, Universitat Autònoma de Barcelona, Barcelona, Spain

**Keywords:** Defiant/Headstrong; developmental trajectories; Irritability; Obsessive-compulsive; Oppositional defiant.

## Abstract

Little is known about the coexistence of oppositionality and obsessive-compulsive problems (OCP) in community children and how it affects their development until adolescence to prevent possible dysfunctions. The co-development of oppositional defiant dimensions and OCP is studied in 563 children (49.7% female) from ages 6 to 13 years, assessed yearly with measures answered by parents and teachers. A 4-class model based on Latent Class Growth Analysis for three parallel processes (irritability, defiant, and OCP) was selected, which showed adequate fitting indexes. Class 1 (*n* = 349, 62.0%) children scored low on all the measures. Class 2 (*n* = 53, 9.4%) contained children with high OCP and low irritability and defiant. Class 3 (*n* = 108, 19.2%) clustered children with high irritability and defiant and low OCP. Class 4 (*n* = 53, 9.4%) clustered comorbid irritability, defiant, and OCP characteristics. The classes showed different clinical characteristics through development. The developmental co-occurrence of irritability and defiant plus obsessive-compulsive behaviors is frequent and adds severity through development regarding comorbidity, peer problems, executive functioning difficulties, and daily functioning. The identification of different classes when combining oppositional problems and OCP may be informative to prevent developmental dysfunctions and to promote good adjustment through development.

## Introduction

Oppositional defiant disorder (ODD) and obsessive-compulsive disorder (OCD) both have their roots in childhood. ODD and OCD may coexist although they are not typically comorbid. All the available information on their association comes from cross-sectional studies with clinical samples of children with OCD who also show behavior problems. It is well known that clinical samples are not representative of the general population as they include individuals with severe symptomatology and a high prevalence of comorbidity. Consequently, there is a gap in the knowledge on the joint manifestation of oppositionality and obsessive-compulsive problems in children from the general population, how these symptoms manifest longitudinally from childhood to adolescence, and which developmental characteristics occur together. This information is essential for prevention purposes and services provision for children from the community. This research aims to fill this gap, obtaining classes of children from the general population that present different degrees of oppositional and obsessive-compulsive symptoms from early childhood to adolescence, and studying relevant developmental characteristics through the 8-year follow-up period. The objectives are framed in the developmental psychopathology paradigm, which promotes the study of the course of the disorders from early life to understand how multiple features of adaptation and maladaptation lead to disorders (Rutter & Sroufe, 2000). The results may help to understand the comorbidity and developmental characteristics of oppositionality and obsessive-compulsive problems when they present together and to identify preventive targets to promote better adjustment in children from the general population.

ODD is characterized by angry mood, disobedience, negativistic and hostile behavior towards authority figures, and vindictiveness. ODD has been conceptualized as a multidimensional disorder with two factors (irritable and defiant) (Burke et al., 2014) or, less frequently, three factors (irritable, defiant, and hurtful) (Wesselhoeft et al., 2019). The irritable dimension includes loses temper, angry, and touchy symptoms; the defiant dimension includes argues, defies, annoys, and blames symptoms (and spitefulness- vindictiveness in the 2-factor solution); and the hurtful dimension includes spitefulness and vindictiveness symptoms. The differentiation of these dimensions has proven to be extremely useful in the clinical setting as they associate with specific disorders, possibly explaining, at least partially, the comorbidities of ODD, which is potentially useful for preventive efforts (Lavigne et al., 2014). For its part, obsessive-compulsive disorder (OCD) refers to the presence of unwanted, nonsensical, recurrent thoughts, urges, or images (obsessions), and/or unnecessary repetitive behaviors or mental acts performed in response to obsession (compulsions) (American Psychiatric Association, 2013). OCD is associated with serious distress and impairment in the daily life of the child and the family (Monzani et al., 2020).

ODD is one of the most prevalent disorders from preschool age, with figures in different countries ranging from 0.4 to 13.4% (Vasileva et al., 2021) and a lifetime prevalence of 10.2% (Nock et al., 2007). ODD shows a moderate continuity through childhood (Husby & Wichstrom, 2017), adolescence (Whelan et al., 2013), and adulthood (Johnston et al., 2018), and is accompanied by severe consequences in academic achievement and social relationships until adulthood (Leadbeater & Ames, 2017). ODD irritability dimension is most often comorbid with anxiety (Martín et al., 2014) and depressive disorders (Burke & Loeber, 2010), and the defiant dimension with attention-deficit/hyperactivity disorder (ADHD) (Harvey et al., 2016) and conduct disorder (Nock et al., 2006). In the case of OCD, in about half of cases the disorder starts in childhood, with prevalence until adolescence ranging from 0.1 to 4% (Heyman et al., 2001), and persistence rates of 40% (Liu et al., 2021). About 80% of children with OCD meet diagnostic criteria for other psychological disorders (Langley et al., 2010). OCD is most often comorbid with depressive and anxiety disorder (Peris et al., 2017), tic disorders (Storch et al., 2008), ADHD (Cabarkapa et al., 2019) and, to a lesser extent, externalizing disorders (Guzick et al., 2019). DSM-5 indicates that OCD is also associated “with disorders characterized by impulsivity, such as oppositional defiant disorder” (p.242).

However, there is no information about the co-development of ODD and OCD from early ages and in community samples. The few available studies on the association of these symptoms have mostly focused on children with OCD and investigate its comorbidity with disruptive behavior disorders. Comorbidity with ODD in children with OCD ranges from 8 to 51% (Peris et al., 2017). In cross-sectional studies, children with OCD and disruptive behavior/externalizing disorders have shown greater OCD symptomatology (Langley et al., 2010), higher functional impairment (Langley et al., 2010), worse quality of life (Storch et al., 2018), greater family accommodation (Storch et al., 2010), and higher anxiety and internalizing problems (Peris et al., 2017) than those without comorbid conditions. There is controversy as to whether this comorbidity is more frequent in younger (Tanidir et al., 2015) or older children (Peris et al., 2017). These studies suggest that the lives of children with both OCD and externalizing problems are severely affected.

Although ODD and OCD may appear to be very different disorders, the reality is that they can coexist, as has been shown in studies on the comorbidity of ODD in samples with OCD. The stubbornness of the oppositional child who wants to do their will and the rituals of the obsessive child who needs to do things in a certain way, the low anger threshold in oppositionism and the anger attacks of the obsessive child when prevented from doing their rituals, the argumentativeness in both cases to be able to do what they want, annoying others for fun or because they need to participate in the ritual, and defying rules may make the two disorders coexist. For example, temper outbursts are common in children with OCD, with about one third of the children diagnosed presenting this symptom (Krebs et al., 2013). Furthermore, youth with OCD and rage attacks presented a higher incidence of disruptive behavior disorders (Storch et al., 2012). The notion of multifinality in developmental psychopathology, which states that common risk factors may lead to different outcomes (Cicchetti & Rogosch, 1996), may explain how different individual and contextual traits may facilitate the co-existence of both symptoms. Executive functioning (McKenzie et al., 2020; Qian et al., 2010), irritability (Stringaris & Goodman, 2009; Theriault et al., 2018), other common comorbidities such as ADHD (Farrell et al., 2020; Harvey et al., 2016) and anxiety (Martín et al., 2014; Storch et al., 2008), and family dysfunction (Greene et al., 2002) may be some of the characteristics that foster overlapping contemporary symptoms of ODD and OCD.

## Current Study

Despite the points made above, little is known about how the coexistence of ODD and OCD may impact the development of affected children and families in the general population. For instance, the natural simultaneous evolution of both symptomatologies in children in the general population, how many of them in the general population suffer from oppositional problems and obsessive-compulsive problems (OCP), and the characteristics that may be more associated with a certain developmental trajectory are unknown. The goal was to study the co-development of oppositional problems and OCP from ages 6 to 13 years in children from the general population and to describe the clinical characteristics of the obtained trajectories. Given that ODD is characterized by two different dimensions the goal was to describe how the dimensions of ODD, irritability, and defiant co-develop with obsessive-compulsive symptoms to find out if a certain dimension or the two dimensions develop simultaneously with obsessive-compulsive problems, and if there should therefore be a specific focus of prevention or treatment. The following research questions are raised. First, how do ODD dimensions (irritability and defiant) and OCP co-develop longitudinally in a general population of children aged 6 to 13 years? And second, what are the clinical characteristics through development associated with the different developmental trajectories obtained. The identification of different classes when combining oppositional problems and OCP may be informative not only for the forecast of future comorbidities, but also for the prevention, assessment, and tailoring of treatment options.

## Methods

### Participants

The sample is part of a longitudinal study of behavioral problems starting at age 3 years described in Ezpeleta et al. (2014). The children (*N* = 2,283) were randomly selected from early childhood schools in Barcelona (Spain). A two-phase design was employed. A total of 1341 families (58.7%) agreed to participate (33.6% high socioeconomic status (SES), 43.1% middle, and 23.3% low; 50.9% boys) in the first phase of sampling. To ensure the participation of children with possible behavioral problems, the parent-rated Strengths and Difficulties Questionnaire (SDQ) conduct problems scale (Goodman, 1997) plus four ODD DSM-IV-TR symptoms (deliberately annoys, blames others, touchy, angry-resentful), not included in the SDQ questions, were used for screening. Two groups were considered: the screen-positive group, which included all the children with SDQ scores ≥ 4, in percentile 90, or with a positive response (*certainly true)* to any of the eight DSM-IV ODD symptoms (*N* = 417; 49.0% boys); and a random draw of children screened negative (*n* = 205; 51.2% boys) (total sample of 622 children aged 3–13 years for the follow-up, mean age = 3.77 years; *SD* = 0.33; 96.9%, born in Spain).

The sample used for this study consisted of 563 children (32.9% screen positive) (Table 1). Because of the age of application of the assessment instruments, data from ages 6 to 13 years (8 assessment points) were used to estimate classes. To this effect, the retention rates in the successive follow-ups with respect to the 563 participants were 85.1%, 83.3%, 76.0%, 78.7%, 75.8%, 79.8%, 64.7%, and 56.7%. No differences were found in sex between the sample at age 6 and the children remaining at age 13 (*p* = 0.903). With respect to socioeconomic status at age 6, the available sample at age 13 had a higher percentage of high SES children (*p* < 0.001).

**Table 1:** Description of the Sample

		At age 6 (*N* = 563)
Age (years); *M* (SD)		6.6 (0.36)
Sex; %	Female	49.7
SES; %	High	33.8
	Medium-High/Medium	46.8
	Medium-low/Low	19.4
Born in Spain; %	Yes	97.1
Ethnicity; %	Caucasian	92.3
	Latino	4.2
	Other	3.5

### Measures

#### Developmental trajectories

##### Dimensions of oppositional problems: Irritability and defiant

The dimension scores of ODD were obtained following Rowe’s (Rowe et al., 2010) 2-factor model. The irritability dimension included three items, ‘touchy-easily annoyed’, ‘angry and resentful’, and ‘loses temper’, and the median (Mdn) of the ordinal alpha in the sample through follow-ups was 0.92. The defiant dimension included five items (‘argues with adults’, ‘defies rules’, ‘deliberately annoys’, ‘blames others’, ‘spiteful’) (Mdn of ordinal alpha = 0.90). These symptoms come from the Strengths and Difficulties Questionnaire (SDQ) (Goodman, 1997) conduct problem scale (loses temper, defies rules, argues, spiteful) plus four questions based on symptoms of DSM-IV ODD not covered by the SDQ and added for the study (deliberately annoys, blames others, touchy, angry-resentful) (0: *not true*; 1: *somewhat true*; 2: *certainly true*). Direct scores for the dimensions were obtained as the sum of the ratings of the corresponding items. Higher scores indicated greater irritability and defiant problems (possible theoretical range of 0–6 and 0–10, respectively). A total score of 2 for both irritability and defiant corresponded to percentile 75 in the sample (equivalent to a total score obtained from the average of the responses to the items of 0.67 and 0.40, respectively; scale: 0–2). For the purposes of this study, scores equal to or higher than percentile 75 in the sample were considered as “high”. Teachers answered the questionnaire every year from when the children were aged 6 to 13 years.

##### Obsessive-compulsive problems (OCP)

The OCP 2007-scale of the Child Behavior Checklist (Achenbach & Rescorla, 2007) contains eight items (9. Obsessions; 31. Fear doing/thinking something bad; 32. Perfectionism; 52. Feels too guilty; 66. Compulsions; 84. Strange behavior; 85. Strange ideas; 112. Worries) (0: *not true*; 1: *sometimes true*; 2: *often true*) that have proven to identify children with clinical problems related to OCD. Parents answered the questionnaire every year from when the child was aged 6 to 13 years (*Mdn* of ordinal alpha = 0.82). The OCP direct score was obtained as the sum of the ratings of the eight items. Higher scores indicated greater problems (theoretical possible range of 0–16). A total score of 1 corresponded to percentile 75 of the sample (equivalent to a total score obtained from the average of the responses to the items of 0.13; scale 0–2). For the purposes of this study, scores equal to or higher than percentile 75 were considered as “high”.

#### Variables through development

If not otherwise specified, all the measures were obtained yearly.

##### DSM-5 diagnoses

The *Diagnostic Interview for Children and Adolescents for Parents of Preschool and Young Children* (DICA-PPYC) (Ezpeleta et al., 2011) is a semi-structured diagnostic interview for assessing DSM-5 psychological disorders. It was answered by the parents at each follow-up. The main diagnoses analyzed were disruptive behavior disorders (ADHD, ODD, and conduct disorder) and anxiety disorders (separation and generalized anxiety, specific, and social phobia). Comorbidity was defined as the presence of more than one disorder among those evaluated in the interview. The presence of any diagnosis, seeking help, and treatment received for any of the diagnoses assessed in the interview were also registered.

##### Dimensional psychopathology

The *Child Behavior Checklist* (CBCL/6-18) (Achenbach & Rescorla, 2001) measures behavioral and emotional problems as reported annually by parents through 112 items with 3 response options (0: *not true*; 1: *sometimes true*; 2: *often true*). Empirical scales plus the dysregulation profile (sum of the items of anxious-depressed, attention problems, and aggressive behavior scales) (Rescorla et al., 2019) were used for the analyses (*Mdn* of ordinal alpha over the eight follow-ups was equal to or above 0.75 for 9 of the 11 scale scores analyzed). Items 31.Fear, 32.Perfectionism, 52.Guilty and 112. Worries on the anxious/depressed and internalizing scales were eliminated to calculate the scores because these items were also included in the OCP 2007-scale. Response categories 1 and 2 (sometimes and often) to items 46 (tics), 58 (picks skin), and 83 (stores up), chosen to indicate OCD-related problems, were grouped to calculate the percentage of presence of these problems.

##### Functional impairment

The *Children’s Global Assessment Scale* (CGAS) (Shaffer et al., 1983) is a global measure of functional impairment rated by the interviewer based on information from the diagnostic interview with the parents at each follow-up.

##### Peer relationship problems

The *Strengths and Difficulties Questionnaire* (SDQ) (Goodman, 1997) assesses children’s mental health with 25 items (0: *not true*; 1: *somewhat true*; 2: *certainly true)* on five scales. The teachers reported on the peer relationship problems scale (*Mdn* of ordinal alpha = 0.82).

##### Temperament

The *Children’s Behavior Questionnaire-Very Short Form* (CBQ-VSF) (age 7) (Putnam & Rothbart, 2006) and the *Early Adolescent Temperament Questionnaire Revised* (EATQR) (age 10) (Ellis & Rothbart, 2001) measure reactive and self-regulative temperament, with 36 items and 62 items (*extremely untrue* to *extremely true*), respectively, on a 7-point and 5-Likert-type scale. It was answered by the parents. The dimensions surgency, negative affect, and effortful control were analyzed (*Mdns* of Cronbach’s alpha were 0.74, 0.81, and 0.79, respectively).

##### Irritability

The *Affective Reactivity Index* (ARI) (Stringaris et al., 2012) contains 6 items about feelings and behaviors related to irritability (0: *not true*; 1: *somewhat true*; 2: *certainly true*) plus one item assessing impairment due to irritability during the last 6 months. The children’s teachers, who had known them for a mean of 10.2 months, answered the ARI questionnaire when the children were 7 and 11 years old, and the child answered the questionnaire at ages 12 and 13 years (*Mdn* of ordinal alpha = 0.85 for teachers’ ratings and 0.95 for the children’s).

##### Executive functioning

The *Behavior Rating Inventory of Executive Function* (BRIEF2) (Gioia et al., 2015) evaluates behaviors showing different domains of executive functioning in daily life. BRIEF2 was used as an outcome measure at the last follow-up to indicate global difficulties in carrying out actions, meeting long-term goals, organizing materials, setting schedules, controlling emotions or impulses, and analyzing or processing information (Gioia et al., 2015). It contains 63 items (0: *never*, 1: *sometimes*; 2: *often*) about behaviors in the last 6 months that reflect how often these behaviors are a problem. It was answered by teachers when the children were 13 years old. The three indexes: the behavior regulation index (inhibit, self-monitor), the emotional regulation index (shift, emotional control), the cognitive regulation index (initiate, working memory, plan/organize, task-monitor, organization of materials), plus the global executive composite (GEC) were used (ordinal alpha values: 0.97, 0.95, 0.99, and 0.99 respectively).

### Procedure

The families were recruited at the schools and gave written consent for the assessment. All the families of the 3-year-old children from participating schools were invited to answer the screening questionnaire. The families who agreed and met the screening criteria were contacted by telephone and interviewed at the school for each annual assessment. The interviewer team was specifically trained, and all the interviewers were blind to the screening group. The teachers answered the questionnaires after permission from the families was obtained.

### Statistical Analysis

The statistical analysis was carried out using MPlus 8.6 and SPSS 24. Given the multistage sampling procedure used, the analyses were weighted by the inverse probability of selection in the second phase of sampling.

Latent Class Growth Analysis (LCGA) for three parallel processes was used to identify distinct groups of individual trajectories considering the direct scores for irritability, defiant, and OCP. As a person-centered approach, LCGA allows individuals with similar patterns to be grouped, focusing on class membership according to time-varying attributes, by obtaining growth parameters such as the intensity of severity (i.e., the initial level) and the amount of growth or decline (i.e., the rate of change or slope) in attributes over time. The Robust Maximum Likelihood (MLR) method of estimation was employed, which enables the inclusion of non-normal and incomplete data, using the expectation-maximization algorithm for missing data with robust standard errors (i.e., full information method). The growth models considered intercept (I), slope (S; i.e., linear trend), and quadratic trend over the eight annual assessments from ages 6 to 13 years, with equal spacing between measurement occasions. The time was rescaled from 6–13 years to 0–7 years, so the first-year assessment (at age 6) represented the intercept.

After checking for possible overlap between measures with bivariate Pearson’s correlations, models with one to six latent classes of growth patterns were obtained. In addition to best clinical interpretability, the following criteria were used to determine the model selected: larger decrement in AIC and sample-size adjusted BIC (aBIC), greater power and more accurate classification by average posterior probabilities, entropy values equal to or greater than 0.70, and more than 5% (*n* > 28) of participants in a class/trajectory. Pairwise mean differences of growth parameter estimates (intercept, slope, and quadratic term) among classes for the selected LCGA model were tested using one-way ANOVA and the Games-Howell correction for post-hoc comparisons, in addition to effect sizes for each comparison (Cohen’s *d*).

Different demographic and clinical characteristics were compared between classes using multiple post-hoc comparisons. To synthesize the information from the follow-ups, a variable was considered as a present for the binary measures if it was present at least one of the follow-ups, while the average of the follow-ups was calculated for the quantitative measures. These summary measures (outcomes) were compared between classes using linear models for the continuous measures, logistic models for the binary ones, and multinomial logistic models for the polytomous measures. The risk of type I error was corrected using Tukey (1949) when comparing the quantitative measures and Bonferroni-Holm’s (Holm, 1979) when comparing the categorical ones.

Internal consistency reliability was calculated using Cronbach’s alpha for questionnaires containing items with 5 or more response options and with ordinal alpha (Elosua & Zumbo, 2008) for items with less than 5 response options.

## Results

Table 2 presents the bivariate correlations between observed scores over waves. Bivariate correlations within each process ranged from 0.22 to 0.63, (irritability: 0.23–0.57; defiant: 0.34–0.63; OCP: 0.22–0.63) and between two processes from −0.07 to 0.82 (irritability-defiant: 0.21–0.82; irritability-OCP: −0.01–0.20; defiant-OCP: −0.07–0.16). Correlation values between observed scores involving two processes cross-sectionally ranged from −0.02 to 0.82 (irritability-defiant: 0.67–0.82; irritability-OCP: 0.00–0.15; defiant-OCP: −0.02–0.15).

**Table 2:** Means, Standard Deviations and Bivariate Pearson’s Correlations between Observed Scores

	*M*	SD	1	2	3	4	5	6	7	8	9	10	11	12	13	14	15	16	17	18	19	20	21	22	23	24
1. Irritability at age 6	1.07	1.38	1																							
2. Irritability at age 7	1.20	1.38	0.57**	1																						
3. Irritability at age 8	1.18	1.46	0.43**	0.43**	1																					
4. Irritability at age 9	1.24	1.43	0.39**	0.42**	0.55**	1																				
5. Irritability at age 10	1.10	1.40	0.43**	0.43**	0.49**	0.49**	1																			
6. Irritability at age 11	1.08	1.38	0.36**	0.35**	0.38**	0.49**	0.53**	1																		
7. Irritability at age 12	1.03	1.45	0.35**	0.37**	0.36**	0.48**	0.43**	0.49**	1																	
8. Irritability at age 13	0.99	1.44	0.23**	0.24**	0.27**	0.29**	0.30**	0.30**	0.44**	1																
9. Defiant at age 6	1.39	1.84	0.74**	0.49**	0.46**	0.40**	0.40**	0.38**	0.34**	0.26**	1															
10. Defiant at age 7	1.45	1.89	0.52**	0.67**	0.44**	0.36**	0.41**	0.34**	0.41**	0.31**	0.63**	1														
11. Defiant at age 8	1.48	2.09	0.45**	0.38**	0.82**	0.45**	0.47**	0.40**	0.37**	0.22**	0.54**	0.49**	1													
12. Defiant at age 9	1.59	1.93	0.41**	0.41**	0.54**	0.70**	0.44**	0.46**	0.45**	0.30**	0.49**	0.45**	0.56**	1												
13. Defiant at age 10	1.63	2.10	0.43**	0.40**	0.46**	0.46**	0.70**	0.50**	0.48**	0.30**	0.48**	0.44**	0.58**	0.55**	1											
14. Defiant at age 11	1.50	2.05	0.37**	0.28**	0.34**	0.47**	0.37**	0.73**	0.46**	0.37**	0.41**	0.38**	0.47**	0.55**	0.59**	1										
15. Defiant at age 12	1.48	2.04	0.34**	0.34**	0.33**	0.44**	0.35**	0.37**	0.72**	0.36**	0.39**	0.41**	0.47**	0.45**	0.57**	0.51**	1									
16. Defiant at age 13	1.55	2.09	0.26**	0.21**	0.28**	0.22**	0.28**	0.30**	0.38**	0.71**	0.38**	0.38**	0.34**	0.36**	0.40**	0.42**	0.45**	1								
17. OCP at age 6	0.84	1.34	0.14**	0.09	0.09	0.06	−0.01	0.08	0.00	0.02	0.08	0.03	0.07	0.08	0.02	0.09	0.08	0.04	1							
18. OCP at age 7	0.84	1.28	0.17**	0.09	0.11*	0.09	0.07	0.06	0.04	0.07	0.11*	0.04	0.07	0.09	0.03	0.02	0.03	0.02	0.50**	1						
19. OCP at age 8	0.88	1.31	0.16**	0.13**	0.15**	0.09	0.08	0.15**	0.10	0.07	0.12*	0.09	0.15**	0.09	0.07	0.11*	0.12*	0.08	0.47**	0.52**	1					
20. OCP at age 9	0.86	1.44	0.18**	0.18**	0.11*	0.09	0.06	0.20**	0.11	0.09	0.12*	0.05	0.09	0.07	0.08	0.11	0.16**	0.03	0.49**	0.44**	0.48**	1				
21. OCP at age 10	0.76	1.24	0.20**	0.19**	0.18**	0.09	0.11*	0.12	0.11	0.07	0.16**	0.12*	0.14**	0.10	0.06	0.04	0.13*	0.01	0.43**	0.46**	0.49**	0.63**	1			
22. OCP at age 11	0.76	1.29	0.13*	0.13*	0.05	0.01	0.06	0.11*	0.04	0.00	0.08	0.08	0.01	0.01	−0.04	−0.02	0.00	−0.07	0.22**	0.37**	0.34**	0.52**	0.59**	1		
23. OCP at age 12	0.57	1.07	0.04	0.09	0.08	0.04	0.00	0.12*	0.00	0.08	0.04	0.05	0.08	0.01	0.00	0.06	−0.02	0.04	0.25**	0.31**	0.35**	0.46**	0.40**	0.55**	1	
24. OCP at age 13	0.59	1.02	0.11	0.16*	0.10	0.06	0.08	0.01	0.03	0.07	0.12	0.03	0.05	0.06	0.02	0.00	0.06	0.04	0.28**	0.30**	0.47**	0.39**	0.46**	0.58**	0.63**	1

*p < 0.05; **p < 0.01; *OCP* Obsessive-Compulsive Problems

### Trajectories of Irritability, Defiant, and OCP

Table 3 shows the goodness-of-fit indices for the LCGA models from one to six classes. Based on the aforementioned criteria, the 4-class model, which showed high entropy (0.892) and very high on-diagonal posterior probabilities of class membership values ( ≥ 0.920), was selected.

**Table 3:** Fitting Indices for 1- to 6- LCGAs Classes for 3 Processes

N. classes	AIC	aBIC	Class: *N* (weighted)	Class: probability*	Entropy
1	36874.1	36912.4	1: 563	–	–
2	34553.8	34603.7	1: 431	1: 0.981	0.921
			2: 132	2: 0.970	
3	33926.1	33987.5	1: 51	1: 0.955	0.918
			2: 116	2: 0.965	
			3: 396	3: 0.966	
**4**	**33368.1**	**33441.1**	**1: 349**	**1: 0.942**	**0.892**
			**2: 53**	**2: 0.941**	
			**3**:**108**	**3: 0.920**	
			**4: 53**	**4: 0.960**	
5	33125.6	33210.1	1:49	1: 0.972	0.906
			2: 345	2: 0.945	
			3: 107	3: 0.924	
			4: 35	4: 0.931	
			5: 27	5: 0.933	
6	32905.4	33001.5	1: 298	1: 0.935	0.884
			2: 36	2: 0.911	
			3: 39	3: 0.918	
			4: 125	4: 0.865	
			5: 27	5: 0.907	
			6: 38	6: 0.968	

*aBIC* Sample-Size Adjusted BIC.

*On-diagonal values for the posterior probability of class membership. In bold: selected solution of LCGA

**Fig. 1:**
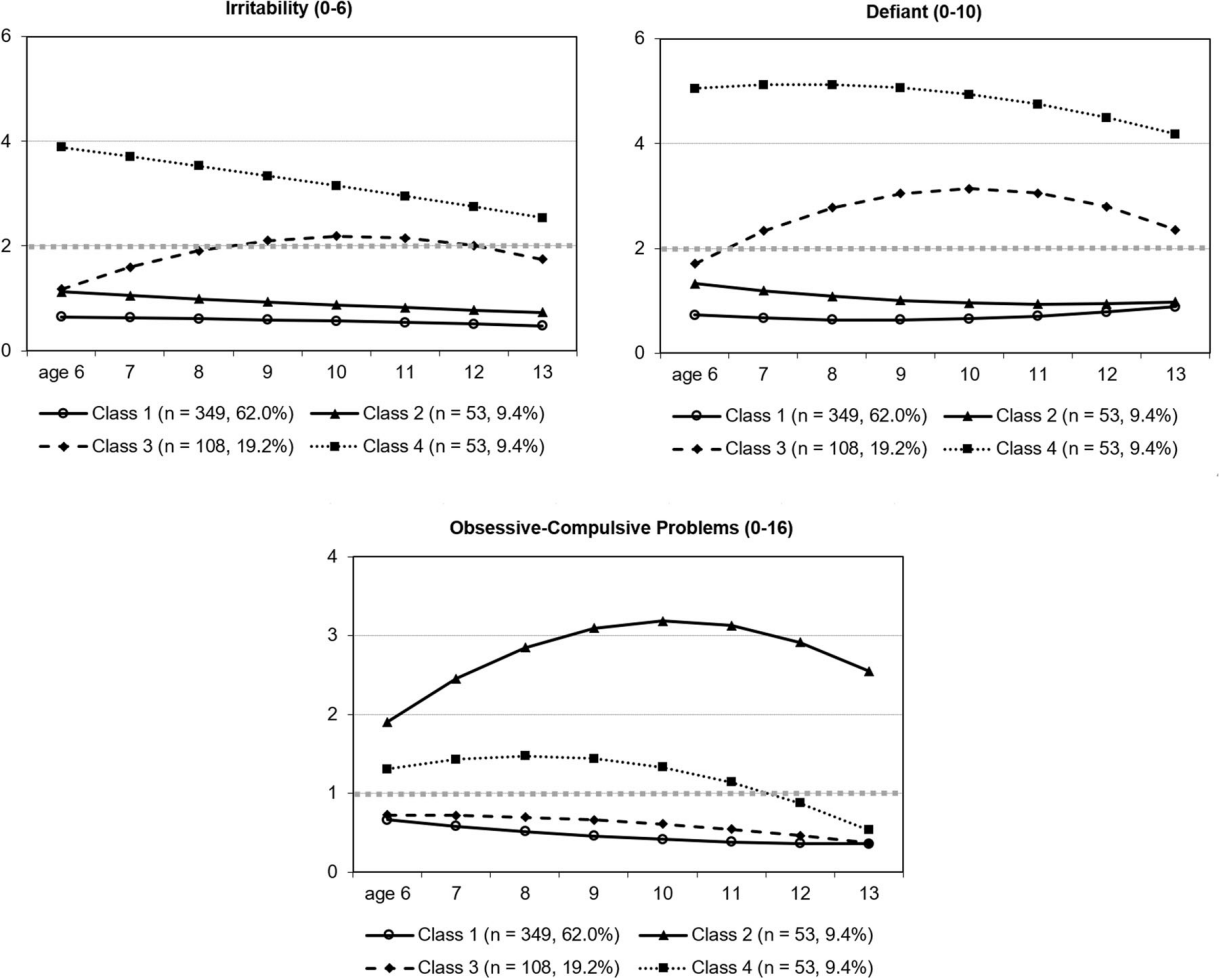
Trajectories for Irritability, Defiant, and Obsessive-Compulsive Problems (OCP) by Processes. Note. Each panel shows the four classes simultaneously for each of the 3 processes over time. The Y-axis corresponds to direct scores based on the sum of item ratings (the title of each figure indicates its theoretical possible scale); the grey horizontal dotted line shows the 75th percentile. Class 1: All low; Class 2: High OCP with low irritability-defiant; Class 3: High Irritability-Defiant with low OCP; Class 4: High Irritability-Defiant-OCP

**Fig. 2:**
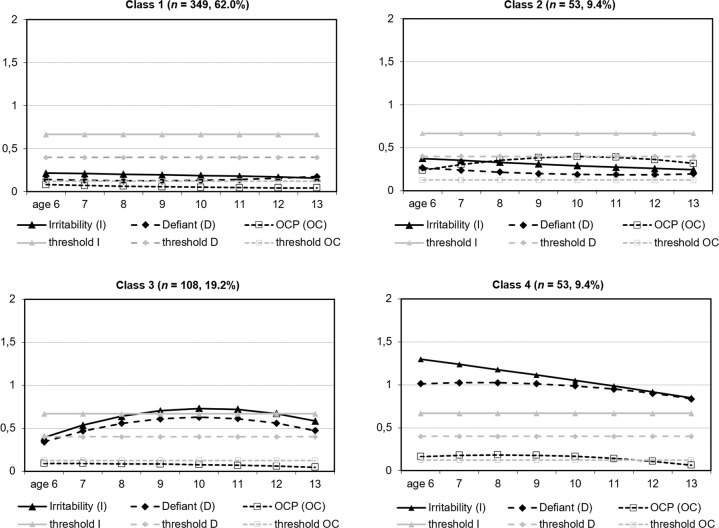
Trajectories for Irritability, Defiant, and Obsessive-compulsive problems (OCP) by Classes. *Note*. Each panel shows the three processes simultaneously for each of the 4 classes. The Y-axis corresponds to the direct scores based on the average of item ratings (0-2 scale for all three processes); the grey horizontal lines show the 75^th^ percentile of the measure with the same line pattern. Class 1: All low; Class 2: High OCP with low irritability-defiant; Class 3: High Irritability-Defiant with low OCP; Class 4: High Irritability-Defiant-OCP

Table 4 presents the parameter estimates for the selected 4-class model, the profiles of which, based on sum-item scores, are shown in Figure 1. Figure 2 represents the three processes jointly for each of the four resultant classes, based on average-item scores (same 0–2 scale for all three processes at Y-axis, also including each corresponding 75th percentile score as threshold). The profile represented in the figures shows that class 1 (*n* = 349, 62.0%) included children with low scores in irritability, defiant, and OCP; class 2 (*n* = 53, 9.4%) included children with high OCP increasing until age 10 years and then decreasing (quadratic trend *p* = 0.020) while maintaining a high score, and low irritability and defiant scores; class 3 (*n* = 108, 19.2%) included children with high irritability scores from age 9 to 12 years (linear trend *p* = 0.002; quadratic trend *p* = 0.015) and high defiant (linear trend *p* = 0.001; quadratic trend *p* = 0.003), but low OCP scores (defiant plus irritability); and class 4 (*n* = 53, 9.4%) included children with high scores in irritability and defiant from 6 to 13 years, and high OCP until age 11, and was labeled as the comorbid class. Both intercept and slope estimates for the three processes involved differed among the four classes (*p* ≤ 0.001; *d* ≥ 0.51). The same was observed for the quadratic trend, except that this parameter for OCP did not differ between classes 1 and 4 (*p* = 0.128). However, the effect size was small but not null (*d* = 0.32 in absolute value) and, as mentioned, both the intercept and the slope did differ. Taken together, we consider there is support for four distinguishable classes (detailed statistics in Appendix).

**Table 4:** Parameter Estimates for the Selected 4-Class Model

Class	Processes	Parameter estimate (*p*)		
		Intercept (basal)	Linear trend (slope)	Quadratic trend
1	Irritability	0.64 (<0.001)	−0.01 (0.712)	−0.00 (0.796)
All low	Defiant	0.72 (<0.001)	−0.07 (0.172)	0.01 (0.076)
	Obsessive-compulsive	0.66 (<0.001)	−0.09 (0.008)	0.01 (0.148)
2	Irritability	1.13 (<0.001)	−0.07 (0.495)	0.00 (0.889)
OCP	Defiant	1.33 (<0.001)	−0.15 (0.354)	0.02 (0.465)
	Obsessive-compulsive	1.91 (<0.001)	0.62 (0.016)	−0.08 (0.020)
3	Irritability	1.18 (<0.001)	0.48 (0.002)	−0.06 (0.015)
Irrit- Defiant	Defiant	1.71 (<0.001)	0.71 (0.001)	−0.09 (0.003)
	Obsessive-compulsive	0.73 (<0.001)	0.00 (0.998)	−0.01 (0.454)
4	Irritability	3.89 (<0.001)	−0.17 (0.420)	−0.00 (0.932)
Irrit-Defiant-	Defiant	5.06 (<0.001)	0.10 (0.702)	−0.03 (0.384)
OCP	Obsessive-compulsive	1.31 (0.001)	0.16 (0.278)	−0.04 (0.067)

OCP Obsessive-compulsive problems

### Clinical Characteristics through Development and Comparisons among the Trajectories

Tables 5 and 6 show the demographic and clinical (categorical and dimensional) characteristics of the four classes obtained through ages 6–13 years and the comparison among classes. The clinical characteristics of each class are briefly summarized as follows. Class 1 (all low) clustered children with the lowest scores in all the variables, the least adverse outcomes, and better functioning in comparison with the other classes.

**Table 5:** Comparisons of Demographic Characteristics and DSM-5 Diagnoses in each Class

	Class 1 All low *n* = 349	Class 2 OCP *n* = 53	Class 3 Irritability- Defiant *n* = 108	Class 4 Irritability- Defiant-OCP *n* = 53	Significant contrasts
*Demographics at baseline*					
Sex (% boys)	45.3	48.1	59.8	65.4	(3 = 4)>1
Socioeconomic status %					n.s.
High	38.0	34.6	31.8	34.0	
Medium/Medium-High	46.4	44.2	44.9	47.2	
Low/Medium-Low	15.6	21.2	23.4	18.9	
*DSM-5 Diagnoses*					
ODD (%)	13.8	37.7	29.6	41.5	(2 = 3 = 4)>1
Subthreshold (%)	46.7	65.4	65.7	67.9	(2 = 3 = 4)>1
Consultation (% Yes)	20.4	33.3	46.2	63.0	4 > (2 = 3)>1
Severity (% Moderate-high)*	18.6	37.7	47.2	69.8	4 > (2 = 3)>1
Family burden (%)*	55.2	73.5	74.7	78.3	(2 = 3 = 4)>1
Treatment (%)*	61.2	81.3	76.2	79.3	n.s.
Age at onset – *M (SD*)	4.3 (2.6)	3.8 (1.9)	4.0 (2.3)	3.3 (1.4)	n.s
ADHD (%)	12.0	28.8	19.4	42.3	4 > 3 > 1; 2 > 1
Age at onset – *M (SD*)	3.3 (1.1)	3.2 (0.9)	3.2 (1.1)	3.4 (1.5)	n.s.
Conduct disorder (%)	0.0	1.9	0.0	7.5	n.s.
Age at onset – *M (SD*)	6.5 (3.3)	5.4 (3.1)	5.3 (2.8)	4.8 (2.5)	n.s.
Any disruptive disorder (%)	18.0	47.2	41.7	59.6	(2 = 3 = 4)>1
Any anxiety (%)	22.6	60.4	23.1	30.2	2 > (1 = 3 = 4)
Any DSM-5 diag. (%)	60.0	92.3	72.9	82.7	(2 = 4)>1; 2 > 3 > 1
Comorbidity DSM-5 (%)	8.6	43.4	15.7	28.3	(2 = 4)>1; 2 > 3 > 1
Consultation any disorder (%)	77.6	86.5	85.2	86.5	n.s.
Treatment by any disorder. (%)	46.2	69.2	63.9	78.8	(2 = 3 = 4)>1
Psychological treatment (%)	21.8	40.4	45.8	65.4	4 > (2 = 3)>1

Linear models for continuous measures; logistic models for binary measures; multinomial logistic models for polytomous measures. Risk of type I error corrected by Bonferroni-Holm’s (Holm, 1979).

*ODD* Oppositional defiant disorder; *ADHD* Attention deficit/Hyperactivity disorder; any anxiety, including separation anxiety, generalized anxiety, specific phobia and social phobia; any disruptive disorder, including ODD, ADHD, CD; comorbidity: presence of more than one DSM-5 diagnosis in the Diagnostic Interview for Children and Adolescents.

*Values calculated among those children with any ODD symptom. *N.s* non-significant

**Table 6:** Comparisons of Dimensional Characteristics, Percentage of Affirmative Response to Items in each Class (mean through follow-ups) and Reliability of the Measures

	α (*Mdn*)	Class 1 All low *n* = 349	Class 2 OCP *n* = 53	Class 3 Irritability- Defiant *n* = 108	Class 4 Irritability- Defiant-OCP *n* = 53	Significant contrasts
*Trajectories variables - M (SD)*						
Irritability-Teachers	0.92	0.6 (0.5)	0.9 (0.8)	1.9 (0.6)	3.4 (1.0)	4 > 3 > 2 > 1
Defiant-Teachers	0.90	0.7 (0.6)	1.0 (0.9)	2.8 (0.8)	5.0 (1.6)	4 > 3 > (1 = 2)
CBC OCP-Parents	0.82	0.5 (0.5)	2.8 (1.0)	0.6 (0.6)	1.3 (1.3)	2 > 4 > (1 = 3)
*Psychopathology - M (SD)* *CBCL 6–18 (T scores)-*Parents						
Anxious/depressed^1^	0.75	47.9 (6.5)	59.2 (10.5)	50.7 (6.5)	54.5 (10.3)	2 > 4 > (1 = 3)
Withdrawn/depressed	0.86	48.7 (7.1)	58.2 (10.6)	49.9 (7.3)	51.9 (10.1)	2 > (1 = 3 = 4)
Somatic complaints	0.74	48.9 (6.5)	58.0 (10.7)	50.9 (8.5)	50.7 (7.3)	2 > (1 = 3 = 4)
Social problems	0.77	47.8 (5.9)	58.7 (10.0)	51.4 (8.0)	55.8 (11.9)	(2 = 4)>3 > 1
Attention problems	0.92	48.2 (8.0)	54.9 (10.3)	53.0 (9.0)	54.6 (9.8)	(2 = 3 = 4)>1
Rule breaking behavior	0.57	48.1 (6.1)	54.2 (10.2)	53.5 (8.8)	57.9 (13.6)	4 > (2 = 3)>1
Aggressive behavior	0.87	47.7 (6.3)	55.4 (11.0)	53.3 (8.4)	58.1 (13.4)	4 > 3 > 1; 2 > 1
Internalizing problems^1^	0.81	48.1 (6.7)	60.8 (10.1)	50.7 (7.4)	53.3 (10.1)	2 > 4 > 1;2 > 3
Externalizing problems	0.88	47.7 (6.3)	55.4 (10.8)	53.6 (8.6)	58.5 (13.5)	4 > 3 > ; 2 > 1
Total problems	0.93	47.4 (6.6)	59.8 (10.2)	52.6 (7.9)	57.1 (12.6)	(2 = 4)>3 > 1
Dysregulation-2007	0.89	47.3 (6.4)	58.8 (10.5)	52.6 (7.6)	57.6 (12.7)	(2 = 4)>3 > 1
*CBCL Items (% of 1–2 response options)*						
46 Tics	NA	26.0	51.9	34.6	42.0	2 > 1
58 Pick skin	NA	25.4	32.1	37.4	32.7	n.s.
83 Stores up	NA	30.5	56.6	39.3	52.0	(2 = 4)>1
*CGAS Functional Impairment- M (SD)*	NA	75.6 (7.0)	67.2 (7.9)	70.1 (7.8)	64.3 (9.9)	1 > (2 = 3); 1 > 4; 3 > 4
*SDQ-*Teachers *- M (SD)*						
Peer problems	0.82	0.9 (0.9)	1.2 (1.0)	1.7 (1.4)	2.5 (1.5)	4 > 3 > (1 = 2)
*Temperament- - M (SD)* *CBQ VSF- EATQ (T scores) -* Ages 7, 10 – Parents						
Surgency	0.74	49.8 (8.7)	45.2 (10.4)	51.6 (7.8)	52.5 (8.9)	(1 = 3 = 4)>2
Negative affect	0.81	47.9 (7.7)	57.5 (9.6)	51.9 (8.7)	54.4 (9.9)	2 > 3 > 1; 4 > 1
Effortful control	0.79	51.3 (7.7)	48.6 (9.3)	47.2 (9.5)	45.7 (10.1)	1 > 3 > 4
*ARI-*Child - Ages 12–13 *- M (SD)*	0.85	1.6 (1.5)	2.1 (1.6)	2.8 (2.2)	2.1 (1.6)	3 > 1
*ARI-*Teacher- Ages 7–11 *- M (SD)*	0.95	0.6 (1.0)	1.1 (1.7)	2.4 (1.6)	4.7 (2.7)	4 > 3 > (1 = 2)
*BRIEF2* (Age 13) *- M (SD)*						
Behavior regulation index	0.97	16.4 (4.5)	15.9 (4.2)	21.2 (6.8)	26.9 (5.8)	4 > 3 > (1 = 2)
Emotional regulation index	0.95	20.0 (4.2)	20.8 (4.9)	23.9 (6.4)	28.0 (6.7)	4 > 3 > (1 = 2)
Cognitive regulation index	0.99	42.5 (13.9)	42.5 (14.4)	50.5 (16.4)	57.2 (12.9)	(3 = 4)>(1 = 2)
Global executive composite	0.99	78.9 (20.2)	79.2 (21.5)	95.6 (25.7)	112.1 (20.1)	4 > 3 > (1 = 2)

Linear models for continuous measures. Risk of type I error corrected by Tukey’s correction (Tukey, 1949) for dimensional comparison and by Bonferroni-Holm’s (Holm, 1979) for percentages comparison. Unless specified table contents direct scores. *N.s.* Non-significant contrast

^1^ Items 31.Fear, 32.Perfectionism, 52.Guilty and 112.Worries were not included in the score of anxious/depressed and internalizing scales because were shared with OCP.

*ARI* Affective Reactivity Index, *BRIEF2* Behavior Rating Inventory of Executive Functions, *CBCL* Child Behavior Checklist, *CBQ-VSF* Children’s Behavior Questionnaire Short Form, *CGAS* Children’s Global Assessment Scale, *EATQR* Early Adolescent Temperament Questionnaire-Revised, *SDQ* Strengths and Difficulties Questionnaire.

Children in class 2 (high OCP with low irritability and defiant) showed a severe developmental trajectory, with a high percentage of DSM-5 diagnoses (92.3%), especially in the any anxiety category (60.4%) and, to a lesser extent, in the any disruptive disorder category (47.2%), high comorbidity (43.4%), high scores (above percentile 90) in the dimensional measures of OCP (measure included in the trajectory), internalizing problems (anxious/depressed, withdrawn/depressed, somatic problems), and negative affect, but low scores in surgency. Parents reported a high frequency (sometimes or often) of tics and storing up.

Class 3 (irritability and defiant) was a class that included mostly boys who, by early adolescence, self-reported high levels of irritability-anger and difficulties in cognitive regulation (BRIEF2 index). The most frequent diagnoses in this class were disruptive behavior disorders (41.7%) (ODD and ADHD).

Class 4 (irritability-defiant and OCP) was the most severe developmental trajectory regarding the different clinical characteristics observed. The most frequent diagnoses in this class were disruptive behavior disorders (59.6%) (ADHD, ODD and conduct disorder). It was made up of mostly boys (65.4%). ODD in this class was moderate-severe, required consultation, psychological treatment, and started at an early age. Children in this class also obtained the highest scores in irritability and defiant (measures included in the trajectory), externalizing symptoms (rule-breaking, aggressive behavior), peer problems, irritability-anger reported by the teacher, and all the scores of executive functioning, and the lowest scores in effortful control. This class presented the worst functioning.

Classes 2, 3, and 4, with high scores in all the processes evaluated, were similar in terms of the percentage of ODD diagnoses (clinical and subthreshold), the associated family burden, the need to seek treatment for the problems, and the scores in attention problems. They all differed from class 1 (all low). Classes 3 (irritability and defiant) and 4 (irritability-defiant and OCP) were similar in terms of the percentage of boys and in the difficulties in executive functions of cognitive regulation. Classes 2 (OCP) and 4 (irritability-defiant and OCP) were similar in the percentage of DSM-5 diagnoses and comorbidity, and in the scores for social and total problems on CBCL.

The association between classes and different psychological characteristics were also analyzed through the first order scales of executive functioning (BRIEF2 questionnaire) and other aggressive behavior scales. This information was discarded due to redundancy.

### Sensitivity Analysis

As a sensitivity analysis, LCGA modelling was replicated using only data corresponding to children with responses for at least 4 of the 8 follow-ups (50%). Global fit indexes suggested that the 4-class solution was optimal, with very similar growth patterns to those observed when analyzing the information using FIML (all children).

## Discussion

Oppositional-defiant and obsessive-compulsive symptoms have proved comorbid in clinical samples in cross-sectional studies, and this coexistence is associated with a more severe clinical picture. However, little is known about how these symptoms coexist and co-develop from childhood to adolescence in community samples. Knowing their developmental paths would be helpful not only to understand the developmental course of boths symptomatologies but also for preventive interventions in the general population. The present study fills this gap through a longitudinal design in which community children were assessed yearly from 6 to 13 years. LGCA with parallel processes identified different classes based on ODD dimensions (irritability and defiant) and OCP scores. A model of four classes fitted the data and clinical interpretability well and showed how irritability, defiant, and obsessive compulsive behaviors co-develop from childhood to adolescence. The four classes represented the individual disorders under study (OCP and oppositional defiant problem dimensions, classes 2 and 3, respectively), and included a comorbid class in which there were high levels of OCP and ODP dimensions (class 4) and a class with low levels of all the characteristics (class 1). The classes showed different clinical characteristics through development. As expected, the comorbid class (class 4) presented a more severe clinical picture where the most marked dimension was irritability, while the pure oppositional problems class (irritability and defiant; class 3) showed stronger externalizing comorbidity and the pure obsessive-compulsive class (class 2) showed stronger internalizing comorbidity plus tics and other obsessive-compulsive related characteristics. These results point to the need for early assessment and follow-up of OCP in children with ODP and the assessment of ODP in children with OCP in community samples. The identification of these classes and their clinical traits may help to better meet the needs of the children in each class.

In about 9.4% of the children in a general population sample oppositional characteristics (irritability and defiant) and obsessive-compulsive behaviors co-develop from ages 6 to 13 years. Most of the previous literature has focused in children with OCD and has warned of a high proportion of comorbidity with ODD (8-51%) and of the need for careful assessment of both conditions (Peris et al., 2017). This study adds to previous data indicating that children in the general population with high scores in oppositionality dimensions may also have high scores in OCP from childhood to early adolescence, also needing careful assessment. In this class, irritability and defiant scores were above percentile 75 in each follow-up, whereas this was the case for OCP from ages 6 to 10, indicating that middle childhood is the developmental period when the co-development of these behaviors is more frequently observed. Irritability tended to diminish with age (although the negative linear trend was not significant) and defiant behavior remained fairly stable through development. Visual inspection of the shape of trajectories in this class shows that the defiant dimension and OCP evolve in parallel, meaning that obsessive-compulsive behaviors (i.e., repeating acts, being perfectionistic, intrusive thoughts, etc.) and defiance (arguing, blaming others, annoying, disobeying) follow the same developmental pattern. While mean scores of OCP in this community sample were generally low, as would be expected, the class that grouped together higher scores in the three processes (irritability, defiant, and OCP) (class 4) showed the worst outcomes through development. In brief, high scores in irritability and defiant (above percentile 75) through development may be accompanied by high scores in OCP (above percentile 75) (or the reverse), and this co-occurrence is associated with externalizing symptoms, functional impairment, peer problems, difficulties in effortful control through development and, by age 13 years, worse general executive functioning. These difficulties were not observed when only irritability and defiant were at high levels and OCP was low (class 3), suggesting a synergistic effect among the three processes when they occur together, which associates with a more difficult development. The results are relevant since it has been reported that the co-occurrence of ODD and OCD in childhood predicts persistence of OCD in early adults (Bloch et al., 2009).

The results on the clinical associations with trajectories point to the role of several possible transdiagnostic variables. According to the Research Domain Criteria approach (Insel et al., 2010), which aims to identify transdiagnostic factors across psychological clinical problems to guide research and clinical practice, deficits in executive functioning seem to be transdiagnostic (East-Richard et al., 2020). However, there is controversy about the implication of executive functions in pediatric OCD and in ODD. While some studies have reported the non-implication of executive functioning in children and adolescents with OCD (Abramovitch et al., 2015), others point to difficulties in action monitoring, decision-making, planning, working memory (Marzuki et al., 2020), organizational skills (Vandborg et al., 2014), and shifting (Lewin et al., 2014). Regarding ODD, while executive function difficulties focus mainly on “hot” executive functions, which are the functions related to emotional involvement (Rubia, 2011), they also include “cool” executive functions, which are those related to tasks requiring cognitive, critical, or logic analysis without the intervention of emotions. Accordingly, difficulties in cognitive reappraisal, rumination, expressive suppression, emotion dysregulation, planning (Jiang et al., 2016), inhibitory control (Deters et al., 2020), working memory and sustained attention (Schoorl et al., 2018), emotion processing, error monitoring, problem solving, and self-control (Noordermeer et al., 2016) have been reported in children with ODD. The results of this study indicate that executive functions difficulties are not characteristic of OCP, since executive functioning is similar in OCP and in the all-low classes (classes 2 and 1), while the four global indexes of executive functioning are significantly higher when there is a confluence of oppositional dimensions at a high level and OCP (class 4). In short, comorbidity exacerbates executive functioning difficulties, suggesting the need to intervene in the improvement of global executive functions in children with oppositional and obsessive characteristics. In the same line, effortful control, or the ability to self-regulate behaviors, cognitions, and emotions, has been proposed as another transdiagnostic dimension for internalizing problems, as would be the case of the comorbidity of OCD, and externalizing problems, as would be the case of ODD and its comorbidity (Santens et al., 2020). In the present study, effortful control was lower in the classes with psychological problems than in the all-low trajectory (class 1) (though the class 2 comparison was not significant), and it had the lowest score in the comorbid class (4), indicating difficulties in self-regulation across problems, which is more accentuated when oppositional dimensions and OCP coexist.

Irritability was the dimension that started with the highest values in comorbid class 4. Irritability in this class decreased through development (the linear trend was not significant) but remained high. Some previous studies have shown the association between anger and OCD. For instance, rage attacks (explosive anger outbursts) were present in 53% of children with OCD and were associated with parental limit setting, need to be perfect, changes in routines and other OCD situations, facilitating reducing irritability and return to calmness (Storch et al., 2012). Anger attacks in Storch’s study were associated with more symptom interference and functional impairment. Similarly, irritability in childhood was associated with OCP and was a strong predictor of obsessive-compulsive behavior in adulthood (Theriault et al., 2018; Theriault et al., 2014). The present and other results in clinical samples point to the need to tackle irritability in cases of oppositional and obsessive-compulsive behaviors.

The classes found represent the problems observed in clinical settings and this gives validity to the classification obtained. For instance, class 2 clustered children with problems on the obsessive-compulsive spectrum who, in addition to OCP scores in the fourth quartile through development, also showed comorbidity with anxiety and depression disorders/symptoms, negative affect, tics and storing up. Children in this class had notable clinical characteristics and functional impairment. In this class OCP increased significantly from ages 6 to 10, and then decreased. This developmental shape may agree with the bimodal distribution of incidence of OCD observed in childhood and adulthood (Boileau, 2011). Class 3, which showed almost parallel trajectories for irritability and defiant throughout development, would be compatible with children with oppositional defiant problems who also showed marked self-reported anger, high comorbidity, and other disruptive behavior problems, such as attention problems and their related cognitive regulation difficulties. A comorbid class also emerged (class 4). This study makes an original contribution to the field by simultaneously analyzing the co-development of irritability, defiant, and OCP from childhood to early adolescence in 8 yearly based follow-ups of a wide sample of boys and girls from the general population. The present study goes beyond previous works, which have mainly focused on the study of externalizing symptoms in children with OCD using variable-centered analyses. Three relevant parallel processes (irritability, defiant, and OCP) reported by different informants (parents and teachers) were able to be analyzed simultaneously using LCGA, modeling heterogeneity by classifying individuals into groups with similar patterns, or latent classes of trajectories. As a result, different classes that reflect the observed phenomenology of oppositional and OCP problems were obtained. The results, however, should be interpreted considering that this was a community sample in which, as expected, psychopathology was not very prevalent. This especially affects obsessive-compulsive behaviors. Such few cases may have implied that differences between measures did not emerge. It should also be considered that the high-low qualification of the labels in the trajectories refers to the levels in the same sample and these may not necessarily be high or low according to normative values, which unfortunately do not exist. Coherence between classes and clinical phenotypes, however, provides criterion validity evidence for the classes obtained. Furthermore, because different contexts (school and home) elicit different behaviors, the study used a multi-informant approach and different reporters to obtain the information (parents, teachers, and child themselves). Although this approach allows us to understand how children display concerning behaviors in the different contexts, it is well known that informants who observe the child in different settings tend to obtain lower levels of correspondence than those reporting about the same setting (De Los Reyes et al., 2015), and this may have decreased the associations. Dimensional measures also show greater levels of cross-informant correspondence than categorical measures (De Los Reyes et al., 2015). Last, more high SES children remained in the study so the results should be generalized with caution. Future research should report on mediational differential paths from trajectories to clinical outcomes that may help to refine preventive interventions to optimize the development of children in the different classes.

The results have preventive implications from a developmental perspective. Middle childhood (6–10 years) is the period of highest irritability, defiant and OCP scores when ODD and OCD coexist. Therefore, it is crucial to develop prevention programs for these ages in community settings such as schools to help manage emotions, and specifically irritability, and to promote cognitive processing that may decrease the OCP and ODD behaviors and their associated clinical characteristics (peer problems, comorbidity, daily functioning). This may prepare the children for the important maturation challenges of adolescence. Adolescence is a period when the brain undergoes marked changes that affect behavior and cognition and is the stage when executive functions start their full maturation. Good adjustment in previous stages may therefore facilitate the transition to adolescence.

## Conclusion

There is a lack of studies in community samples on how oppositional and obsessive-compulsive symptoms codevelop from childhood to adolescence and their associated characteristics through development. This gap makes it difficult to design preventive strategies that facilitate optimal development in children in the community. Several classes reflecting different developmental trajectories of oppositional defiant dimensions and OCP from childhood to adolescence were identified. Coherence between classes and clinical phenotypes may provide criterion validity evidence for the classes obtained. The co-development of oppositional defiant dimensions (irritability and defiant) and OCP is frequent, affects about 9.4% of the children aged between 6 and 13, and is associated with psychological difficulties throughout development. Children in the comorbid trajectory presented a more severe externalizing symptomatology and comorbidity with ADHD, used psychological services more frequently, had higher functional impairment and difficulties with peers and effortful control, had higher scores in anger/irritability and, by adolescence (age 13), displayed generalized difficulties with executive functions. Clinicians should be aware that ODD and OCP may coexist, and that this coexistence is associated with compromised development. Therefore, faced with oppositional defiant problems or OCP, an appropriate assessment of both conditions and a good differential diagnosis is necessary. In children sharing oppositional and obsessive-compulsive characteristics, preventive strategies targeting executive functioning, effortful control, peer relations, and irritability may be indicated to facilitate better adjustment during development.

## Appendix

*Comparison of Parameter Estimates for the Selected 4-Class Model*

**Table:**

Measures	Growth	Overall test		Means (SD)				Comparison: 1 vs 2			Comparison: 1 vs 3			Comparison: 1 vs 4			Comparison: 2 vs 3			Comparison: 2 vs 4			Comparison: 3 vs 4		
Process	Parameter	*F* (*df*)	*p*	class 1	class 2	class 3	class 4	*MD*	*p*	*d*	*MD*	*p*	*d*	*MD*	*p*	*d*	*MD*	*p*	*d*	*MD*	*p*	*d*	*MD*	*p*	*d*
Irritability	I	7775.0 (3. 558)	<0.001	0.68 (0.08)	1.11 (0.07)	1.20 (0.20)	3.78 (0.26)	−0.43	<0.001	−5.23	−0.52	<0.001	−4.20	−3.10	<0.001	−25.61	−0.09	0.001	−0.51	−2.67	<0.001	−14.39	−2.58	<0.001	−11.70
	S	2264.0 (3. 558)	<0.001	0.00 (0.05)	−0.06 (0.02)	0.44 (0.08)	−0.15 (0.06)	0.07	<0.001	1.61	−0.43	<0.001	−7.85	0.15	<0.001	3.23	−0.50	<0.001	−7.59	0.08	<0.001	1.88	0.59	<0.001	7.92
	Q	2174.0 (3. 558)	<0.001	0.00 (0.01)	0.00 (0.00)	−0.05 (0.01)	−0.01 (0.01)	0.00	<0.001	−0.95	0.05	<0.001	8.09	*0.00*	*0.128*	*0.32*	0.05	<0.001	7.90	0.01	<0.001	1.69	−0.05	<0.001	−6.51
Defiant	I	7607.2 (3. 558)	<0.001	0.78 (0.13)	1.31 (0.09)	1.71 (0.27)	4.92 (0.32)	−0.53	<0.001	−4.12	−0.93	<0.001	−5.34	−4.14	<0.001	−24.54	−0.40	<0.001	−1.79	−3.61	<0.001	−15.66	−3.21	<0.001	−11.35
	S	2302.6 (3. 558)	<0.001	−0.04 (0.07)	−0.14 (0.03)	0.65 (0.11)	0.12 (0.06)	0.10	<0.001	1.44	−0.69	<0.001	−8.23	−0.16	<0.001	−2.30	−0.79	<0.001	−8.42	−0.26	<0.001	−5.73	0.53	<0.001	5.41
	Q	2473.0 (3. 558)	<0.001	0.01 (0.01)	0.01 (0.00)	−0.08 (0.01)	−0.03 (0.01)	0.00	<0.001	−0.53	0.09	<0.001	8.38	0.04	<0.001	4.72	0.09	<0.001	8.26	0.05	<0.001	10.72	−0.05	<0.001	−4.00
Obs-Comp.	I	3756.8 (3. 558)	<0.001	0.69 (0.07)	1.83 (0.15)	0.75 (0.07)	1.29 (0.05)	−1.15	<0.001	−13.66	−0.06	<0.001	−0.89	−0.60	<0.001	−8.92	1.09	<0.001	10.51	0.55	<0.001	4.89	−0.54	<0.001	−8.18
	S	3518.8 (3. 558)	<0.001	−0.07 (0.04)	0.58 (0.09)	0.01 (0.03)	0.15 (0.01)	−0.65	<0.001	−13.36	−0.08	<0.001	−1.98	−0.23	<0.001	−5.86	0.58	<0.001	10.41	0.43	<0.001	7.02	−0.15	<0.001	−5.38
	Q	3685.2 (3. 558)	<0.001	0.00 (0.00)	−0.07 (0.01)	−0.01 (0.00)	−0.04 (0.00)	0.08	<0.001	13.11	0.01	<0.001	2.54	0.04	<0.001	8.90	−0.06	<0.001	−9.85	−0.03	<0.001	−4.73	0.03	<0.001	8.01

*I* Intercept, *S* Slope, *Q* Quadratic trend. In italics: pair-wise comparison with *p* > 0.05

## Abbreviations

OCD: Obsessive-compulsive disorder
OCP: Obsessive-compulsive problems
ODD: Oppositional defiant disorder
ODP: Oppositional defiant problems
